# Effectiveness of a SCERTS Model-Based Intervention for Children with Autism Spectrum Disorder (ASD) in Hong Kong: A Pilot Study

**DOI:** 10.1007/s10803-018-3649-z

**Published:** 2018-06-15

**Authors:** Lu Yu, Xiaoqin Zhu

**Affiliations:** Department of Applied Social Sciences, The Hong Kong Polytechnic University, Hunghom, Hong Kong, People’s Republic of China

**Keywords:** SCERTS model, Quasi-experiment, Evaluation, Chinese children with ASD, Focus group

## Abstract

A SCERTS model-based intervention with different durations (5-month vs. 10-month) was provided to 122 children with autism spectrum disorder (ASD) (age = 53.43 ± 9.05 months) in Hong Kong. Before and after the intervention, the children were assessed with the Chinese Psychoeducational Profile-Third Edition (CPEP-3) and the Developmental Assessment Chart (DAC). Educators and parents expressed their views toward the intervention in focus groups. Results showed that participating children improved significantly in their social communication and emotional behavior after the intervention, as measured by DAC and CPEP-3. Likewise, educators and parents had positive views toward the intervention and noted the children’s improvement. The results suggest that a SCERTS model-based intervention can improve social communication, emotional regulation, and other skills in children with ASD.

## Introduction

Children with autism spectrum disorders (ASD) demonstrate significant impairment in their social interactions and communication and exhibit a restricted range of interests and attention (American Psychiatric Association [APA] 2013). These deficits interfere with their learning and disrupt their family life. There is considerable agreement in the field of ASD research that intensive and early intervention leads to significant improvements in these children’s functioning and long-term outcomes (National Research Council [NRC; USA] 2001). The complicated needs of children with ASD and their families require not only support from schools, teaching staff, educators, and specialized workers, but also consistency in the intervention approaches and strategies used by those different parties. Without adequate collaboration, the parties that work with children with ASD may have their own individual, respective focuses and specific approaches in intervention and may use different assessment tools to gather the same information. Those differences may result in inconsistent interventions, repetitive assessments, and fragmented services that act on parts of a child instead of addressing the child as a whole person (Prizant et al. 2005). These issues will in turn negatively affect the well-being and outcomes of children with ASD and their families (Barnes 2008; O’Reilly et al. 2013). To optimize intervention implementation and maximize long-term achievement, it is contended that a comprehensive educational model should be used to guide early intervention for children with ASD, for the purpose of ensuring “child-centered” and “family-centered” practices as well as of addressing the need for consistency and accountability (Prizant et al. 2003).

The SCERTS model is a comprehensive and multidisciplinary educational approach (Prizant et al. 2003) that aims to enhance the social communication (SC) and emotional regulation (ER) of individuals with ASD through the implementation of transactional support (TS). Two notable characteristics of the model are worth highlighting. First, the model is in line with evidence-based practices, in that the design of the model draws from a significant amount of empirical research evidence (Prizant et al. 2003). Specifically, the two major focuses of the model, social communication and emotional regulation, echo the most critical priorities in promoting the development of life skills among children with ASD (National Research Council [NRC; USA] 2001). It is believed that enhancing social communication and socioemotional competencies in children with ASD will promote their ability to access a purposeful and meaningful education and achieve better long-term outcomes (Rubin et al. 2013).

Second, the model coheres the related partners around the child (e.g., family, professionals, peers, community) and improves multidisciplinary teamwork (O’Neill et al. 2010). On one hand, family-professional partnerships set a platform for providing individualized service to improve the well-being of children with ASD and their families. On the other hand, communication and collaboration among teachers and specialized workers minimize, and may even completely eliminate, a fragmented approach (Hayes 2015), thereby leading to implementing evidence-based interventions consistently and comprehensively. In turn, such consistent and comprehensive interventions help the children and families to achieve the most positive long-term outcomes. To conclude, the SCERTS model uniquely emphasizes multidisciplinary teamwork, and empirical findings have suggested that using the SCERTS model supports professionals in their implementation of an educational plan for children with ASD by promoting teamwork and exchanging beneficial daily practices (Molteni et al. 2013).

It is believed that interventions based on the SCERTS model brings about positive impacts not only on children with ASD but also on the related partners (e.g., parents, special education teachers, and therapists) (Prizant et al. 2005). Therefore, the model has been widely used in Western countries, such as the USA, the UK, and New Zealand. However, current evaluative studies of the effectiveness of the SCERTS model, with regard to its impacts on children with ASD, are far from sufficient. Only few exceptions can be identified. For example, a pilot case study involving four pupils with ASD in a primary special school in the UK showed that, after one year of intervention with the SCERTS model, all four pupils had made significant progress in the areas of joint attention, symbol use, and mutual and self-regulation, as reflected by their behavioral scales test scores and the observations of the teaching staff (O’Neill et al. 2010). Another case study, in New Zealand, found that music therapy incorporating the SCERTS model enabled a 3-year old boy with ASD to improve across socioemotional growth indicators, such as active learning and organization, social membership and friendship, independence and cooperation, and appropriateness of behavior (Ayson 2011). More recently, Wetherby et al. (2014) conducted a randomized controlled trial to examine the effects of a SCERTS model-based parent-implemented intervention. The results showed significant improvement in the individual home coaching group in terms of the participating children’s social communication, adaptive behavior, and developmental level, and those improvements supported the effectiveness of parent-implemented intervention.

Compared with the wide use of the SCERTS model in Western countries, its application in training children with ASD in the Chinese context has been limited. There is also a dearth of research examining the model’s effectiveness in helping Chinese children with ASD. In Hong Kong, the Heep Hong Society introduced the SCERTS model in 2011, and the society has since then incorporated it into providing services to local children with ASD. However, they implemented the SCERTS model on a small scale, and the impacts of the practice have not been systematically investigated. To fill that gap, the Heep Hong Society has commenced a project that aims to adapt and implement the SCERTS model on a larger scale and has combined that project with a research study to assess the effectiveness of the project in helping children with ASD, their parents, and their teachers. While some prior studies found that longer treatment duration had positive effects on the effectiveness of the treatment for children with ASD (Linstead et al. 2017; Virués-Ortega 2010), some concluded that treatment duration alone was not a strong enough predictor of the effectiveness of the intervention (Virues-Ortega e al. 2013). On the basis of previous research findings, the project also considered intervention duration as a factor that may influence the effectiveness of the intervention program and included two treatment groups with different treatment durations (i.e., 5-month treatment vs. 10-month treatment). This paper reports the preliminary evaluative findings of that project, using a mixed-method research design.

## The Present Study

The objective of the present study was to investigate the effectiveness of using a SCERTS model-based intervention as a framework in training preschool children with ASD in Hong Kong. To comprehensively examine that effectiveness, the current study attempted to address three research questions.

Research question 1: Did child participants’ performance significantly improve after their participation in the SCERTS model-based intervention? We expected the children to have significantly higher scores on multiple developmental outcomes, especially in the domains of social communication and emotional regulation (e.g., communications skills, emotional behavior, etc.) after the intervention. The outcomes measured in the present study will be described with more details in the “assessment tools” section below.

Research question 2: Did duration of the intervention influence the effectiveness of the SCERTS model-based intervention? Due to inconclusive findings in previous research regarding the effect of treatment duration (Linstead et al. 2017) and the lack of literature pertaining to the SCERTS model-based intervention, we did not make a specific hypothesis for this research question.

Research question 3: What were the opinions of the intervention implementers (teachers and therapists) and parents of the participating children, with regard to the implementation of the SCERTS model-based intervention and its impact? We expected the implementers and parents to have positive views toward the intervention’s implementation and its impact on the participating children and other parties involved.

## Method

### Overview of the Project

The project was launched by the Heep Hong Society, and 65 educators (including 34 special education teachers, 10 speech therapists, 10 occupational therapists, and 11 physiotherapists) from 10 special childcare centers in Hong Kong were recruited to participate. All of the participating educators were well-educated, with a bachelor’s degree or above in social work, psychology, or special education. Most of them were experienced in working with children with ASD and had a minimum of 3-year related working experience.

**Fig. 1:**
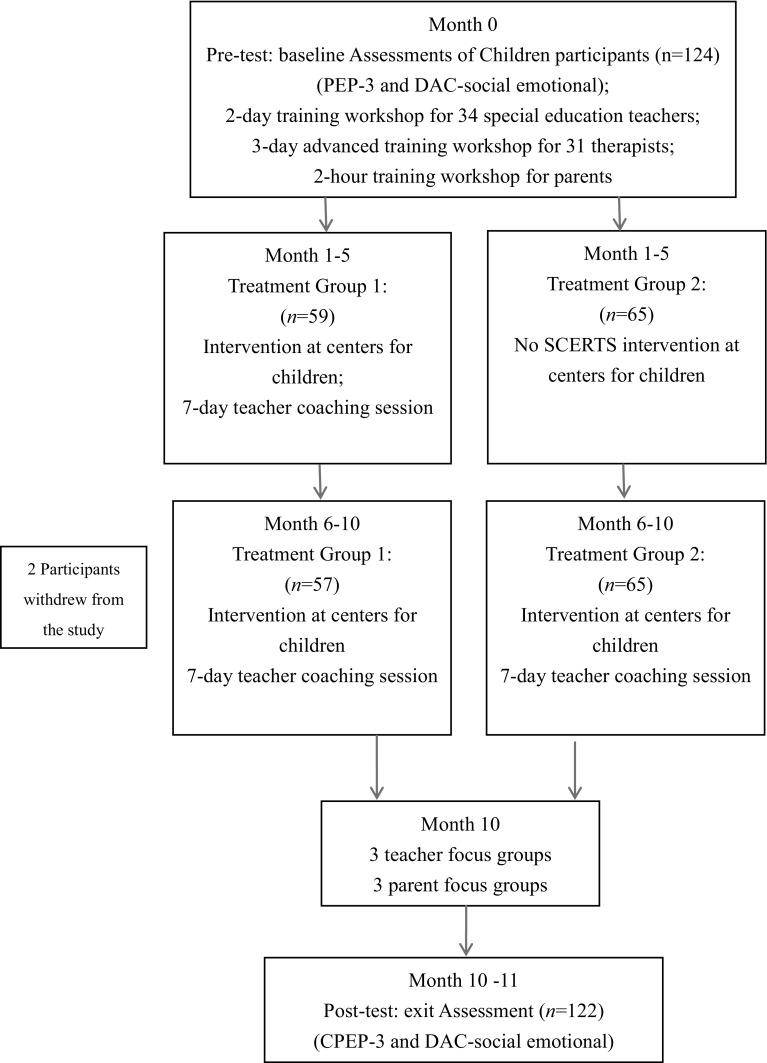
Procedures of the project

Fig. 1 depicts the procedure of the project. Prior to the start of the SCERTS model-based intervention, these 65 educators entered an intensive training program that was designed to help them acquire knowledge and skills about implementation of the intervention for children with ASD. The training program had two components: (1) a 2-day training workshop on the SCERTS model, designed for all the 34 special education teachers and (2) a 3-day advanced training workshop on the SCERTS model, for all the 31 therapists. In addition, a 2-h training workshop that introduced the SCERTS model and its application in the daily lives of children with ASD was provided for the parents of the children in the 10 centers, in order to help them understand the SCERTS model’s rationale and basic ideas. Educators and parents attended the respective intensive training workshops together (see Fig. 1).

After the training, all participating educators implemented either a 10-month or 5-month SCERTS model-based intervention in their respective centers. There were 18 special education teachers, 5 speech therapists, 5 occupational therapists, and 5 physiotherapists in the 10-month group and there were 16 special education teachers, 5 speech therapists, 5 occupational therapists, and 6 physiotherapists in the 5-month group. Each special education teacher taught five to seven children with ASD and/or another developmental delay. Therapists provided weekly individual therapy and group treatment session for children (with the group size ranged between 4 and 6 children) in the center.

During the intervention period, two measures were taken to further ensure and monitor the quality of the program’s implementation. First, a 14-day coaching session and a 7-day coaching session (both with 6 h of observation and 2 h of case discussion each day) on incorporating the SCERTS model into intervention practices for children with ASD, were provided for the 18 and 16 special education teachers in the 10-month and 5-month groups, respectively. The coaching sessions were conducted separately for two groups of teachers spreading over the implementation period. Specifically, special education teachers received one- or two-day coaching every month in their respective centers (see Fig. 1). Second, three times during the intervention period a total of eight trained professionals from the Heep Hong Society observed the SCERTS model-based intervention being implemented by the participating educators at each center. The monitoring professionals used a 22-item quality indicator scale to rate the extent to which each indicator was met in the program’s implementation, in terms of program planning, implementation, monitoring, and adjustment, and also the extent of transactional support, including both learning and interpersonal support. Each item was rated with a 4-point Likert scale, with 0 = no or minimal evidence that this is happening, 1 = this is happening some of the time (less than 50%), 2 = this is happening most of the time (more than 50%), and 3 = this is always happening (i.e., more than 90% of the time). An average score across corresponding items were calculated as the score of each quality indicator. When there was more than one monitoring professionals in the same class, the average score of their ratings were used. On the basis of the rating scores, the educators were given oral feedback and suggestions to help them make an action plan to address the identified shortcomings of the indicator in question. Mean quality scores of participating educators ranged from 1.22 to 2.91 for the 10-month intervention (with a group average of 2.32), and from 1.91 to 3.0 for the 5-month intervention (with a group average of 2.44), thus suggesting good intervention quality for both intervention durations.

### Participants and Procedures

The present study utilized quantitative and qualitative evaluative designs, with the quantitative design addressing the first two research questions and the qualitative design addressing the third research question. For the quantitative part, a quasi-experimental pre- and posttest design was employed. Specifically, 124 children with ASD in 10 special childcare centers were recruited to participate in the study. Children were invited if: (1) they had a clinical diagnosis of ASD, (2) they were from 3 to 6.5 years in age (i.e., preschool age), and (3) their primary caregivers gave informed consent.

Among the 10 centers, five centers were randomly selected and assigned as Group 1. Fifty-nine children in Group 1 received a 10-month SCERTS model-based intervention, with 35 h of training per week including one weekly individual therapy session (45 min) and one weekly group therapy session (60 min with 4–6 children in each group). The remaining five centers were assigned as Group 2, and the 65 children in this group received a 5-month SCERTS model-based intervention, again with 35 h of training each week and weekly individual therapy session and group therapy session. The 5-month intervention was implemented 5 months after the 10-month intervention started. All the 10 special child care centers offer full-day intensive training and care service 5-days-a-week. Parents of participants agreed to send their children to the center every weekday unless with specific reasons (e.g., sickness, or extreme weather). No report was received from the child centers regarding the absence of any participants during the intervention period, suggesting that all participating children completed the required training.

All participating children did not receive any other interventions during the study period (i.e., 10 months). A group of trained professionals from the Heep Hong Society who were not involved in the intervention and were blind to the study’s objectives used standardized assessment tools to rate the child participants in both treatment groups, prior to the implementation of the 10-month intervention (i.e., pretest) and after the completion of all intervention treatments (i.e., posttest).

During the intervention period, two children in Group 1 dropped out of the study due to a change of their childcare center, which left 122 child participants (96 boys, age = 53.43 ± 9.05 months at posttest) in the final analyses. More specifically, the present analyses were based on 57 children (45 boys, age = 53.28 ± 8.95 months at posttest) in Group 1 and 65 children (51 boys, age = 53.55 ± 9.21 months at posttest) in Group 2. There were no significant differences regarding gender composition or age between the two groups, either on the individual level or the center level.

To better understand the effectiveness of using the SCERTS model, the present study also included a qualitative evaluation to address the third research question. After the conclusion of the SCERTS model-based interventions, parents of the child participants and educators who implemented the interventions were also invited to participate in focus group interviews to share their views and experiences about the implementation and impact of the interventions. For each group, we invited 10 parents and 10 educators to participate. A total of 19 parents (10 from the 10-month group and 9 from the 5-month group) attended three focus group interview sessions (n = 6, 9, and 4, respectively), and 20 educators (10 from the 10-month group and 10 from the 5-month group) attended another three focus groups (n = 6, 5, and 9, respectively). For both types of focus group interviews (i.e., parents or educators), participation rates of the two treatment groups were favorable and did not differ significantly from each other. All interview sessions were conducted by external postgraduate student researchers trained in the SCERTS model, and each session lasted for 1.5 to 2 h.

A focus group interview guideline was used to facilitate the interviews. Specifically, we invited the educators and parents to express their views and experiences by asking the following three questions: (1) what were their perceptions of the SCERTS model, (2) what changes had they observed in the participating children after attending the program, and (3) what were the impacts of the SCERTS model on their own attitude, knowledge, and skills for training children with ASD. All interviews were audio-recorded upon the participants’ consent.

Procedures of the present study are depicted in Fig. 1.

### Assessment Tools

In the present study, standardized assessment tools utilized in the pre- and posttests consisted of two measures, as described below.

#### Chinese Psychoeducational Profile-Third edition (CPEP-3)

Translated from the PEP-3 developed by Schopler, Lansing, Reichler, & Marcus (2005), the CPEP-3 is a validated assessment tool that comprehensively measures the development of Chinese children with ASD (Shek and Yu 2014, 2015). The questionnaire includes a performance test and a caregiver report. The performance test was administered by trained professionals from the Heep Hong Society who were blind to the study’s aims, and it was based on direct testing and observation following a standard procedure. The performance test was composed of 10 subtests: (1) cognitive verbal/preverbal (34 items), (2) expressive language (25 items), (3) receptive language (19 items), (4) fine motor (20 items), (5) gross motor (15 items), (6) visual-motor imitation (10 items), (7) affective expression (11 items), (8) social reciprocity (12 items), (9) characteristic motor behaviors (15 items), and (10) characteristic verbal behaviors (11 items). Specifically, subtests 1 through 3 measured communication skills, subtests 4 through 6 measured motor ability, and subtests 7 through 10 measured maladaptive behaviors.

The caregiver report (38 items) was completed by the child’s primary caregiver and was based on daily observation of the child’s performance in three dimensions, including problem behavior, personal self-care, and adaptive behavior. Consistent with previous studies (Shek and Yu 2014), all subtests showed acceptable to good internal consistency in the present study, with Cronbach’s alpha coefficients ranging from 0.71 to 0.84.

#### Developmental Assessment Chart

The revised version of the Developmental Assessment Chart (Heep Hong Society 2013) assesses children’s development across a wide variety of domains, including gross motor skills, fine motor skills, language, cognition, social-emotional skills, and self-care skills. This scale has been used as an educational assessment tool that identifies children’s relative strengths and weaknesses. Besides, it can also be used to monitor children’s developmental progress in the six domains for the purpose of educational planning. In the present study, the children’s developmental conditions on these items were also rated by trained professionals from the Heep Hong Society who were blind to the study’s objectives, and used a scale ranging from 0 (never) to 2 (always). In this study, two subscales relevant to ASD and the treatment targets were used, including the Social Communication Skill Scale and the Emotional Behavior Scale. We used the two subscales to investigate whether the children participants had any improvement in these two domains after the treatment. The Social Communication Skill Scale measured positive social functioning (19 items) and the Emotional Behavior Scale measured the children’s understanding, expression, and regulation of emotions (20 items). The DAC doesn’t provide standardized scores, therefore, raw scores of the two subscales were utilized. The Cronbach’s alpha coefficients for the two scales at pre- and posttest were all above 0.75 for both groups.

### Data Analyses

A MANOVA was first conducted posttest to examine the equivalence of the two treatment groups, in terms of the children’s age and development, at both individual and center levels. Specifically, for the individual level, group comparison regarding each outcome indicator was based on the average score across participants in corresponding group. For the center level, average scores of participants in each center regarding all outcome indicators were calculated as the center-level aggregated scores, which were then compared between the two group.

Second, a mixed ANOVA with pre- and posttest scores as repeated measures (i.e., within-subjects factor) and treatment group (i.e., Group 1 and Group 2) as the between-subjects factor was separately performed for each outcome indicator, to examine the students’ development after the training while controlling the potential effects of baseline conditions.

Third, multiple regression analyses were carried out to examine potential effects of the intervention’s duration. The children’s posttest score on each outcome indicator was treated as the dependent variable, and age, the related pretest score, and the group (1 = 10-month intervention; 0 = 5-month intervention) were inputted simultaneously as the independent variables. One regression analysis was performed for each outcome measure. Significant effects of the group on posttest scores would suggest that program duration may influence the program’s impact on children’s outcomes.

The six audio recordings for the educator and parent focus group interviews were transcribed into full texts for further thematic analyses, and to ensure data accuracy the transcriptions were carefully checked by two individuals from the research staff. Following the guidelines established for qualitative analysis (e.g., Wolcott 1994) and previous practices in analyzing qualitative data (e.g., Shek et al. 2014), the transcriptions were analyzed using a thematic analysis method. Because the purpose of this qualitative analysis was to address a specific evaluation question, emerging themes were identified in a deductive manner on the basis of the interview question asked (i.e., perceptions of the SCERTS model, children’s changes, and impact on educator’s attitude, knowledge and skills of training children with ASD).

First, all transcriptions were reviewed and reorganized into three data sets, each of which corresponded to one of the three interview questions. Second, each data set was carefully reviewed several times to identify content themes that could provide important information regarding the interview question. Third, conceptually similar content themes were categorized into the same major theme. For example, content themes that related to the special education teachers’ perceptions of changes in their roles and the methods used in working with children with ASD were grouped together and formed a major theme called “impact on teaching practice.”

To calculate inter-rater reliability, 20 narratives were randomly selected and coded by a trained research assistant who was not involved in the current study and did not know the originally derived themes. The inter-rater reliability was 90%, calculated in terms of agreement percentage regarding emerged major themes of coding. Researchers further discussed inconsistent coding to reach an agreement on major themes. For all representative quotations presented in the "Results" section below, standardized translation and back-translation procedures were employed to ensure the authenticity of the narratives.

## Results

### Baseline Comparisons

Participating children’s age and pretest scores on the DAC and CPEP-3 subscales were first compared between the two groups at the individual level and the center level. Results of the MANOVA are shown in Table 1. At the individual level, Group 2 (the 5-month intervention group) appeared to have better performance than Group 1 (10-month intervention) did in terms of social communication (*F* = 23.08, *p* < .001, *η*^*2*^ = 0.16), emotional behavior (*F* = 24.05, *p* < .001, *η*^*2*^ = 0.17), receptive language (*F* = 6.38, *p* < .05, *η*^*2*^ = 0.05), visual-motor imitation (*F* = 4.48, *p* < .05, *η*^*2*^ = 0.04), and problem behavior (*F* = 6.41, *p* < .05, *η*^*2*^ = 0.05). On age and other outcome indicators, the two groups did not differ significantly from each other at baseline.

**Table 1:** Pretest comparison between the two intervention groups

Assessment variables	Individual level						Center level					
	Group 1 (*n* = 57)		Group 2 (*n* = 65)		MANOVA		Group 1 (*n* = 57)		Group 2 (*n* = 65)		MANOVA	
	*M*	*SD*	*M*	*SD*	*F*	*η* ^2^_*p*_	*M*	*SD*	*M*	*SD*	*F*	*η* ^2^_*p*_
Age (months)	53.43	9.05	53.55	9.21	0.03	0.0002	52.69	5.15	53.87	2.76	0.20	0.02
Developmental assessment chart												
SC	14.23	5.60	19.49	6.40	23.08***	0.16	14.64	2.68	19.62	3.20	7.10*	0.47
EB	15.12	4.70	20.00	6.08	24.05***	0.17	15.68	2.44	20.14	2.90	6.91*	0.46
CPEP-3 performance test raw score												
CVP	33.49	13.82	37.03	13.39	2.06	0.02	34.58	5.97	38.24	7.00	0.79	0.09
EL	9.51	8.85	12.52	10.37	2.94	0.02	10.12	3.92	13.16	6.05	0.89	0.10
RL	15.53	9.95	20.06	9.85	6.38*	0.05	16.16	3.43	20.70	5.15	2.70	0.25
FM	29.44	6.19	30.49	5.95	0.92	0.01	29.87	2.64	31.07	3.19	0.42	0.05
GM	22.77	6.19	24.29	5.27	2.15	0.02	22.99	2.48	24.62	2.33	1.14	0.13
VMI	11.16	4.07	12.86	4.48	4.78*	0.04	11.41	1.90	13.21	2.20	1.93	0.19
AE	12.81	3.77	12.92	4.33	0.03	0.0002	12.82	1.28	12.99	2.03	0.02	0.00
SR	12.35	4.41	13.45	4.38	1.88	0.02	12.62	1.68	13.66	2.41	0.63	0.07
CMB	19.93	5.43	21.65	6.13	2.65	0.02	19.87	2.16	22.10	3.19	1.67	0.17
CVB	8.19	6.07	8.71	6.49	0.20	0.002	8.36	1.79	8.94	3.12	0.13	0.02
CPEP-3 caregiver report raw score												
PB	7.05	4.11	8.82	3.58	6.41*	0.05	6.87	1.67	8.87	2.13	2.74	0.26
PSC	14.25	5.21	15.37	3.92	1.84	0.02	14.38	0.82	15.54	1.74	1.82	0.19
AB	15.44	5.71	17.02	4.82	2.73	0.02	15.53	2.48	17.13	2.42	1.06	0.12

Group 1, 10-month intervention; Group 2, 5-month intervention; SC, Social Communication; EB, Emotional Behavior; CPEP-3, Chinese Psychoeducational Profile-Third Edition; CVP, cognitive verbal/preverbal, EL, expressive language; RL, receptive language; FM, fine motor; GM, gross motor; VMI, visual-motor imitation; AE, affective expression; SR, social reciprocity; CMB, characteristic motor behaviors; CVB, characteristic verbal behaviors; PB, problem behavior; PSC, personal self-care; AB, adaptive behavior

* *p* < .05, *** *p* < .001

At the center level, the two groups did not differ significantly on age or most of the outcome indicators, with social communication (*F* = 7.10, *p* < .05, *η*^*2*^ = 0.47) and emotional behavior (*F* = 6.91, *p* < .05, *η*^*2*^ = 0.46) being two exceptions. For those two indicators, Group 2 showed better performance than Group 1 did.

### Comparisons of Pre- and Posttests

Comparisons of the pre- and posttests as repeated-measures and treatment group (10-month intervention vs. 5-month intervention) as between-subjects variable are shown in Table 2. For the DAC scores, results showed that children in both groups performed significantly better in the posttests than in the pretests, in terms of social communication skills and emotional behavior (*p* < .001 for both), with large effect sizes being indicated by the partial *η*^2^ values (i.e., greater than 0.4).

**Table 2:** Quantitative results of pretest and posttest comparisons regarding the children’s performance

Assessment variables	Group 1 (*n* = 57)				Group 2 (*n* = 65)				Main effect of Time		Time × Group	
	Pretest		Posttest		Pretest		Posttest					
	*M*	*SD*	*M*	*SD*	*M*	*SD*	*M*	*SD*	*F*	*η* ^2^_*p*_	*F*	*η* ^2^_*p*_
Developmental assessment chart^a^												
SC	14.23	5.60	18.81	5.86	19.49	6.40	21.86	7.44	116.74***	0.49	11.81**	0.09
EB	15.12	4.70	19.67	6.09	20.00	6.08	22.85	7.48	79.91***	0.40	4.22	0.03
CPEP-3 performance test raw score^b^												
CVP	33.49	13.82	44.67	15.06	37.03	13.39	47.60	14.79	272.04***	0.69	0.21	0.002
EL	9.51	8.85	16.58	11.37	12.52	10.37	20.03	14.17	152.21***	0.56	0.14	0.001
RL	15.53	9.95	23.53	9.96	20.06	9.85	26.43	9.63	210.11***	0.64	2.71	0.02
FM	29.44	6.19	34.16	5.24	30.49	5.95	35.22	4.36	218.58***	0.65	0.00004	0.00
GM	22.77	6.19	27.00	4.05	24.29	5.27	27.42	3.61	108.98***	0.48	2.46	0.02
VMI	11.16	4.07	14.44	4.00	12.86	4.48	16.15	3.74	181.19***	0.60	0.001	0.00
AE	12.81	3.77	14.58	3.86	12.92	4.33	15.45	4.71	76.90***	0.39	2.35	0.02
SR	12.35	4.41	14.82	4.52	13.45	4.38	17.18	4.86	104.42***	0.47	4.33	0.04
CMB	19.93	5.43	22.26	5.44	21.65	6.13	23.85	6.06	52.55***	0.31	0.05	0.0004
CVB	8.19	6.07	9.89	6.15	8.71	6.49	11.12	6.69	58.62***	0.33	1.76	0.01
CPEP-3 care giver report raw score^c^												
PB	7.05	4.11	7.88	4.38	8.82	3.58	8.91	4.44	2.35	0.02	1.50	0.01
PSC	14.25	5.21	15.46	4.69	15.37	3.92	17.15	5.41	13.96***	0.10	0.51	0.004
AB	15.44	5.71	15.75	6.64	17.02	4.82	17.62	6.77	0.99	0.008	0.10	0.001

Group 1, 10-month intervention; Group 2, 5-month intervention; SC, Social Communication; EB, Emotional Behavior; CPEP-3, Chinese Psychoeducational Profile-Third Edition; CVP, cognitive verbal/preverbal, EL, expressive language; RL, receptive language; FM, fine motor; GM, gross motor; VMI, visual-motor imitation; AE, affective expression; SR, social reciprocity; CMB, characteristic motor behaviors; CVB, characteristic verbal behaviors; PB, problem behavior; PSC, personal self-care; AB, adaptive behavior

* *p* < .05, ** *p* < .01, *** *p* < .001

^a^Adjusted Bonferroni value = 0.03

^b^Adjusted Bonferroni value = 0.005

^c^Adjusted Bonferroni value = 0.02

With respect to the CPEP-3 subscale scores, the children in both groups also performed better in posttests than they did in pretests, in terms of all performance subtests (*p* < .001 for all), and with all partial *η*^2^ values exceeding 0.3. For caregiver report scores, the children participants in both groups showed significantly better scores at posttest for only one (i.e., personal self-care) out of the three dimensions. In other words, children did not show significant improvement at other two dimensions (i.e., problem behavior and adaptive behavior) after the treatment.

Overall, mixed ANOVAs showed that the children in both groups performed significantly better on the posttests than on the pretests in terms of social communication and emotional behavior, as measured by the DAC, and in communication skills, motor ability, and adaptive behaviors, as assessed by the CPEP-3 (*p* < .001 for all). partial *η*^2^ values were all above 0.3, thus indicating large effect sizes. Therefore, our hypothesis on the first research question (i.e., children would have significantly higher scores on multiple assessment tools after the intervention) was supported.

### Influence of Program Duration

As shown in Table 2, treatment group as the between-subjects factor had significant interactions with repeated measures for only 1 out of 15 outcome indicators, i.e., social communication (*F* = 11.81, *p* < .01, *η*^2^_*p*_ = .09). The result suggested that the impacts of treatment duration (group) on participants’ progress were minimal.

To further confirm the potential influence of program duration, we also performed regression analyses in which the effects of program duration (1 = 10-month duration vs. 0 = 5-month duration) on the participants’ posttest scores were examined after controlling for their pretest scores. Results are shown in Table 3. The results show that program duration (group) did not significantly predict posttest scores on 13 out of 15 outcome indicators. For social communication (*β* = 0.15, *p* < .01, Cohen’s *f*^*2*^ = 0.07) assessed by the DAC, and social reciprocity (*β* = −0.16, *p* < .01, Cohen’s *f*^*2*^ = 0.06) assessed by the CPEP-3, the effects of program duration were significant but in different directions, and the effect sizes were not big. Longer program duration had a positive association with social communication, whereas it had a negative association with social reciprocity, on the posttests.

**Table 3:** Prediction of age, pretest score, and program duration on posttest scores

DV	*β*_Age_	*β*_Pretest score_	*β*_Group_	Cohen’s *f*^*2*^ for group	*R*^2^ change
DAC					
SC	0.03	0.91***	0.15**	0.07	0.74***
EB	0.01	0.80***	0.10	0.02	0.59***
CPEP-3					
CVP	− 0.13**	0.88***	0.01	0.00	0.78***
EL	− 0.10*	0.87***	0.001	0.00	0.78***
RL	− 0.17***	0.85***	0.04	0.01	0.75***
FM	− 0.20***	0.86***	− 0.04	0.01	0.71***
GM	− 0.09	0.76***	0.05	0.00	0.55***
VMI	− 0.15***	0.79***	− 0.07	0.01	0.66***
AE	− 0.14**	0.78***	− 0.09	0.02	0.66***
SR	− 0.19**	0.72***	− 0.16**	0.06	0.60***
CMB	− 0.12*	0.82***	− 0.02	0.00	0.70***
CVB	− 0.13**	0.87***	− 0.06	0.02	0.82***
PB	− 0.15*	0.69***	0.04	0.00	0.50***
PSC	− 0.15*	0.63***	− 0.09	0.01	0.38***
AB	− 0.09	0.66***	− 0.04	0.00	0.46***

DAC, Developmental assessment chart; Group: 1, 10-month intervention; 0, 5-month intervention; SC, Social Communication; EB, Emotional Behavior; CPEP-3, Chinese Psychoeducational Profile-Third Edition; CVP, cognitive verbal/preverbal, EL, expressive language; RL, receptive language; FM, fine motor; GM, gross motor; VMI, visual-motor imitation; AE, affective expression; SR, social reciprocity; CMB, characteristic motor behaviors; CVB, characteristic verbal behaviors; PB, problem behavior; PSC, personal self-care; AB, adaptive behavior

* *p* < .05, ** *p* < .01, *** *p* < .001

In short, above-mentioned findings seem to suggest that the length of the intervention (5 months vs. 10 months) has little or inconclusive influence on the effectiveness of the program.

### Focus Group Interviews

Five major themes were identified from the focus group interviews with educators and parents, with the first four major themes deriving from interviews with both parties and the last one theme deriving from interviews with educators.

#### A Comprehensive and Multidisciplinary Model

The first main theme arose from the interviews with educators and parents on the question of their “perception of the SCERTS model,” and both parties related the theme to their perceptions of the model’s comprehensiveness in addressing the core challenges of children with ASD. The educators and parents both regarded the SCERTS model to be a well-designed training model with clear objectives, assessment methods, and intervention and evaluation procedures. It measured the children’s individual developmental needs and determined tailor-made support efforts to be provided by the children’s social partners. The concepts of emotional regulation and transactional support were seen as being new and useful to teachers, because those concepts helped them better understand the children’s needs and interests. Following are some example narratives from the educators and parents:

It’s a very comprehensive model that helps us to evaluate and understand our students in terms of not only their deficits in specific developmental areas, but their strengths and needs. This is important for us to make individualized educational objectives and design appropriate activities for students to participate in. (One educator’s sharing)

“The training [of the SCERTS model] reminds me to pay more attention to my child’s emotions and behaviors, and more importantly, to interpret these behaviors in a new way. When I understand more about the reasons behind [the behaviors], I can better respond to him, and help him to learn how to express his needs properly. (One parent’s sharing)

Although educators and parents both regarded the SCERTS model as comprehensive, educators also perceived the SCERTS model as multidisciplinary. The educators perceived the model as being flexible enough to incorporate other practices from approaches such as the Treatment and Education of Autistic and Communication Handicapped Children (TEACCH) (Schopler et al. 1995) and the Developmental Individual-difference Relationship-based model (DIR) (Wieder and Greenspan 2005; Greenspan and Wieder 1997). Because the SCERTS model valued the input of a team of professionals, all of the professionals, including the teachers and therapists, were involved in the SCERTS process to formulate and implement an individual education plan for each child with ASD and to discuss the whole class transactional support. Some teacher participants further shared that they worked more closely with the parents in that process, and the narratives below are some examples:

“To enable our students to function well in different social settings, we need a closer collaboration among different professionals and with parents. We used to consider parents’ roles as mainly [that of] a follower, but now we have tried to involve parents in different stages of our work, from the initial assessment to educational planning, implementation, and monitoring.” (One educator’s sharing)

#### Improvement in the Children’s Social Communication

On the basis of the interview with educators and parents on the question of “changes in the children,” the second major theme that emerged relates to the improvement in the children’s social communication. Educators and parents both observed the children’s improved performance in the area of social communication, including in joint attention, social initiation, communication, and interaction. Following are some example narratives:

“He [the child] started to pay more attention to people and things around [him], for example, what his peers were doing. He also used more gestures, such as pointing to indicate his needs.” (One educator’s sharing).

“Communication cards as a means of visual support helped increase children’s intent to communicate with others and make requests. If I put the cards away, my student seldom requested help but sat at his place and waited for help. With the use of visuals, he learned that he could show me the “helping” card as a way to request help. After 3 months, he would seek help from teachers when needed, without any prompts. This was a very big improvement for him.” (One educator’s sharing).

“Before the implementation of the SCERTS model, my daughter rarely shared her thoughts with me, due to her limited speech… One day, I saw her using the cards to form the sentence, ‘I go to toilet’ on the communication board and I was really surprised that she had made such big progress. My child learnt to use the sentence board at home and form short phrases using the cards.” (One parent’s sharing).

“He showed more interest in his peers; for example, he would smile at them and hug them. His parents also shared that he interacted more with them and his siblings at home.” (One educator’s sharing).

Discussion also pointed to additional positive outcomes observed by educators and parents, such as the children’s increased confidence in their expression and communication. After implementation of the SCERTS model-based intervention, some children were more fluent in their speech and used a clearer tone of voice in communication.

#### Improvement in the Children’s Emotional Regulation

The third major theme, which is related to the children’s improved emotional regulation, also emerged from the interview with educators and parents on the question of “changes in the children.” In terms of emotional regulation, both educators and parents indicated that most children showed a better understanding of basic emotions and, with the assistance of educators and parents, learned to use behavioral or language strategies to express their emotions. A few children were able to generalize what they learned and to self-regulate their emotions at home. Views toward the children’s development in emotional regulation are reflected in the quotations below:

“My child is very responsive to visual supports, and he likes to read the emotion cards. Now he [has] learnt to smile when happy and close his eyes to calm himself down. When he was upset, he would express his emotions using the cards, which helped me understand more about him and better respond to him, for example, by giving him a hug.” (One parent’s sharing)

“He used to hit me when things did not go his way. I expressed the feeling of pain on my face, with the use of emotion cards. He seemed to understand more about how I feel even though the concept was quite abstract to him. Now he would stop hitting me when I told him it was painful.” (One parent’s sharing)

“I used my body language and the emotion card to teach my daughter the emotion of fear. Once she understood the concept, I taught her that when she was scared, she could seek adults’ help or asked them to hug her. One day, in a restaurant, someone suddenly spoke with a very loud voice, which scared her. I saw her hugging herself to calm herself down. I was really happy to see her applying what she learnt, in real life situations.” (One parent’s sharing)

Apart from the children’s improved emotional regulation, improvement in their arousal level and attention was also observed. Some of the children who had a low arousal level were able to regulate their state with the use of behavioral support, such as holding sensory toys or chewing. Special education teachers reported that the children were able to stay engaged in activity for a longer period of time; for example, the period of engagement increased from 1 min before to 7 min after the intervention. For children with better verbal skills, some learned to express their feelings, such as tiredness, and to ask for a short walk or permission to lie on a beanbag to help restore their arousal level. With the use of such regulatory support, the teachers commented that although the children spent some time regulating their own arousal level in class, the teachers also spent less time on managing the children’s inattentive behaviors.

#### Impact on the Educators’ Attitudes

The fourth major theme emerged from the interviews with educators and parents on the question of “the impact of the SCERTS model on their own attitude, knowledge, and skills.” Responses of educators and parents indicated that their involvement in the SCERTS project shifted how they thought about and worked with children with ASD. A few special education teacher participants shared that initially they had doubted the feasibility of applying the SCERTS model in classroom setting. However, those attitudes changed with the implementation of the SCERTS model-based training. For instance:

I used to believe that children should be well-disciplined. They were asked to sit still and look at the teacher. It was hard for me to let them walk around the classroom as a way to regulate their arousal…After understanding the meaning behind – addressing children’s needs, I started to change my mindset.

Before I learned the SCERTS model, I expected children to do what was told in class all the time. Now I observe their needs and interests more to see what they like to do first. The atmosphere of the lesson has become more relaxed and interactive.

Other areas of impact that educators and parents shared included more acceptance of the children’s challenging behaviors, and a higher sense of self-efficacy. The majority of educators and parents started to reflect on how to better work with children with ASD and meet their developmental needs. Given better understanding of the children’s developmental stages and the capabilities associated with each stage, educators treated “problem behavior” as not “just” the child’s problems. Instead, they interpreted problem behavior as meaningful and honored protests, whenever appropriate and possible. The educators and parents also adopted a more positive approach in order to provide the necessary level of support to the children.

#### Impact on Teaching Practice

The last major theme emerged from responses of the educators, especially the special education teachers of them, to the question of “the impact of the SCERTS model on their own attitude, knowledge, and skills.” The educators indicated that their roles changed when they were provided with the SCERTS model-based intervention. They shifted their roles from being highly directive to being more facilitative in their training of children with ASD. Areas of change included (1) offering more choices, and waiting and facilitating shared control between educators and children; (2) providing more visual and organizational supports, to encourage the children’s participation and transition across activities; and (3) modifying the goals, activities, and learning environment to promote initiation of communication and emotional regulation. Some special education teachers shared that:

My lesson was highly teacher-directed as I decided what children needed to do. After the implementation of the SCERTS model, children were given more choices to choose [from] and they could decide what they wanted to do first.

The teaching timetable used to be highly packed and more than three activities were organized in a 30-min lesson. After I started the SCERTS model, I reduced the number of activities and slowed down the teaching pace so that children were given enough time to respond and participate.

Instead of me doing the teaching all the time, I put in more social communication elements; for example, designing more interactive games, to facilitate children’s interaction with their peers.

The experience of implementing the SCERTS model-based training also encouraged the special education teachers to provide the children with a more diversified learning experience in which they could engage more actively. With the use of visual supports, the children showed better understanding of the structure of the lesson and their role in learning and exhibited less problem behavior as a result. The learning atmosphere became more relaxed, with less tension between the teachers and children. More focus was put on promoting the children’s social communication and emotional regulation, and that laid the foundation for learning and development.

Overall, both educators and parents had positive views of the application of the SCERTS model, observed significant improvement in the participating children in multiple areas, and thought that their involvement in the SCERTS model-based intervention positively influenced their own attitude toward training children with ASD. Therefore, our hypothesis on the third research question was also supported.

## Discussion

The present study is the first to evaluate the effectiveness of SCERTS model-based training in helping preschool children with ASD in a Chinese context, and it was based on a mixed-method design. The results revealed that after the intervention, participating children showed significant improvement in multiple domains of their development, as reflected in objective assessments and also in subjective reports by educators and parents that were based on their observations and reflections. Educators and parents also expressed that the SCERTS model helped them better understand the needs and interests of children with ASD, changed their attitudes and roles in their daily practices, and enabled them to provide more effective support to the children. These findings substantiated the usefulness of incorporating the SCERTS model into designing and providing interventional and educational services to Chinese children with ASD.

As is the case with most of the currently available comprehensive treatment models for children with ASD (Arick et al. 2003; Handleman and Harris 2006), the SCERTS model has been theory-based and well operationalized but has yielded limited evidence on the treatment outcomes (Lin et al. 2016; Odom et al. 2010). Researchers have published manuals that provide detailed guidance for users on how to assess the child, plan and develop appropriate programs, and implement intervention (Prizant et al. 2005). However, very few studies have examined the effectiveness of the model, particularly in terms of child outcomes, and no previous study was conducted in a Chinese context. The present findings fill those gaps by showing the impact of the SCERTS model-based intervention on children’s developmental outcomes in a sample of preschool children with ASD in Hong Kong. Children receiving the training demonstrated substantial improvement in areas that are related to the core deficits in ASD; that is, in social communication and emotional regulation. The quantitative findings are corroborated by qualitative results obtained from the interviews with educators and parents. Given the paucity of evidence on the effects of comprehensive treatment models on child outcomes (Odom et al. 2010), particularly in a different cultural context, these findings are uniquely important.

In terms of the duration of the program, in almost all measured areas (14 out of 15 outcome indicators), treatment duration did not significantly interact with the intervention effects (i.e., changes from pretest to posttest). Besides, the effect sizes for intervention effects were moderate to large. The multiple regression analyses also confirmed that program duration had limited predicting effects on posttest scores after the pretest scores were statistically controlled. All these findings suggest that the 5-month intervention appeared to have similar impacts to those of the 10-month intervention on the participating children’s development. Previous literature has suggested that although treatment intensity has significant and reliable positive effects on participants’ improvement, the effect of treatment duration has been inconclusive (Linstead et al. 2017). In the present study, both the 5-month training and the 10-month training offered 35 h of active engagement with the participants every week, and that high treatment intensity is likely to have contributed to the significant changes shown in both groups, regardless of the program’s duration. On the other hand, the findings may also suggest that the impact of the SCERTS model-based intervention can be observed in a relatively short period of time. These observable changes may further encourage parents and educators to continue to adopt the SCERTS model-based approach in their daily practices. It should be noted that the 5-month intervention group performed better than the 10-month intervention group did on five indicators in the pretests. Although no significant differences were found for other developmental areas, it is possible that this group of children had a relatively higher developmental level and were more receptive to the intervention than the 10-month intervention group was. Although the potential influence of pretest scores was statistically controlled, the current results should still be interpreted with caution.

Although the SCERTS model focuses specifically on social communication skills and emotional regulation abilities of children with ASD, the participating children also demonstrated significant development in other areas, such as motor ability and personal self-care. For example, in both training groups, the children’s performance on fine motor skills, gross motor skills, and visual-motor imitation significantly improved after the training, with large effect sizes (*η*^2^_*p*_ > .48 for all). Caregivers also reported improved personal self-care in their children after they had attended the program. These findings are also consistent with the sharing by parents and educators in the focus group interviews. The additional child outcomes of the intervention may be attributed to the enhanced transactional support, another key element of the SCERTS model. As was reported by parents and educators in the interviews, they were guided to respond to the needs and interests of the children, to modify the environment, and to use tools to facilitate the children’s learning during the training. Emotional and educational support provided to educators and parents further improved their confidence in promoting the children’s development and coping with problems encountered. This speculation is reasonable, because the prior research also found that transactional support contributed not only to the participating children’s development in the key deficit areas, but also to their overall development (Molteni et al. 2013).

Strengths of the present study are the mixed-method design, the use of valid and reliable objective measures for child outcomes, and the inclusion of two treatment groups, who received 5-month and 10-month SCERTS-based training, respectively. Objective assessments of participating children’s development were made by both professionals and parents. Such findings are less likely to be influenced by the expectations of parents and teachers on the program’s effects. The comparison of the two intervention groups provided preliminary information about the effect of program duration. In addition, in focus group interviews, educators and parents all expressed positive views toward the SCERTS-based trainings, in terms of the comprehensiveness of the model, the children’s improvements in multiple areas, and the positive impacts of the intervention on their own attitude and practices. These qualitative findings not only triangulate the quantitative findings but also demonstrate that incorporation of the SCERTS model in training children with ASD will benefit children, their parents, and professionals.

Several limitations of the present study should be acknowledged. First, although we adopted a pretest and posttest design to examine the changes of the participating children before and after the program, there was no control group, and that limited the interpretation of the findings. We cannot rule out the possibility that the improvements observed in both groups were due to natural maturation rather than to the SCERTS model-based training. Randomized control trials (RCTs) need to be undertaken in the future to examine how the program could contribute to the positive changes in children, after excluding the effects of maturation. In addition to a blank control group, a normal treatment control group without incorporating the SCERTS model could also be included in the RCT, and that would help researchers discern whether the changes in the participants were due to the normal treatment or to the SCERTS model-based treatment. The effects of program duration (5 months vs. 10 months) should also be further tested in RCT studies.

Second, because parents received training on the SCERTS model before the SCERTS model-based intervention, the influence of parental practices cannot be clearly differentiated from the program’s effects. Data gleaned from the focus group interviews suggested that parents played an important role in enhancing the effects of the intervention program. Future study should directly measure parental practices and examine their mediation effects on child outcomes.

Third, we relied on children’s previous clinical diagnosis of ASD to screen eligible children participants. As we did not know the reliability of those diagnosis and each children’s autism symptom severity, it will be better if future studies apply independently confirmed ASD diagnosis for selecting participants. In addition, the participating children’s cognitive development level was not measured in the present study, and future research must take that potential confounding factor into account when examining the effects of a SCERTS model-based intervention.

Fourth, all participating children were considered achieving the intervention goal of 35-h training per week as no report was received from the children centers regarding the absence of participating children during the intervention period. However, to better monitor the intensity of intervention, future studies will benefit from having a clear attendance record of participating children and calculating each child’s actual training participation.

Fifth, we did not apply specific standard to examine educators’ performance before they started the intervention, as all educators were well-educated and experienced in working with children with ASD. However, they might still differ from each other regarding their understanding and skills of implementing a SCERTS model-based intervention, which may affect the outcome of the intervention. Such potential influence can be minimized by the random assignment of participating centers into one of the two treatment groups. Nevertheless, it will yield more robust results if future research also control for educators’ performance.

Last, but not least, the present study was based on a relatively small sample of children with ASD in Hong Kong, a special administrative area of China. There are cultural differences between Hong Kong and other cities of China that might affect the implementation of a SCERTS model-based intervention. For example, whereas Cantonese is used as the spoken language in Hong Kong, the majority population in China speaks Mandarin. Therefore, the effects of a SCERTS model-based intervention should be examined in a broader Chinese context, on the basis of a more representative sample of children with ASD.

Despite those limitations, the present study provides preliminary empirical support to the beneficial effects of the SCERTS model-based intervention for Chinese children with ASD. The study’s positive findings have practical implications for the following situations in Hong Kong. First, researchers and practitioners in Hong Kong who are working with Chinese children with ASD may wish to consider further incorporating the model into their existing treatment/educational programs, and examining the program’s impacts with regard to experimental design. Second, professionals can also provide regular training for parents in order to support them and help them further apply this model in their daily lives in an effort to help their children with ASD. Third, professionals working with children with ASD can effectively collaborate with each other on the basis of this model. Collaborative practices that are based on the SCERTS model, when employed by teachers, social workers, and psychologists, will benefit children with ASD and their families in the long run. In addition, such practices will also bring positive changes to the various treatment partners in terms of their knowledge, attitudes, and skills when they are working with children with ASD.
